# Chlorhexidine gluconate lavage during total joint arthroplasty may improve wound healing compared to dilute betadine

**DOI:** 10.1186/s40634-022-00503-w

**Published:** 2022-07-10

**Authors:** Brandon E. Lung, Ryan Le, Kylie Callan, Maddison McLellan, Leo Issagholian, Justin Yi, William C. McMaster, Steven Yang, David H. So

**Affiliations:** 1 Department of Orthopaedic Surgery, University of California Irvine Medical Center, 101 The City Drive South, Pavilion 3, Orange, CA 92868 USA; 2 School of Medicine, University of California Irvine, Irvine, CA 92617 USA

**Keywords:** Betadine, Chlorhexidine, Lavage, Arthroplasty, Infection

## Abstract

**Purpose:**

Intraoperative wound irrigation prior to closure during total joint arthroplasty (TJA) is an essential component of preventing infections and limiting health care system costs. While studies have shown the efficacy of dilute betadine in reducing infection risk, there remains concerns over its safety profile and theoretical inactivation by blood and serum. This study aims to compare infection and wound complications between chlorhexidine gluconate (CHG) and betadine lavage during TJA.

**Methods:**

All primary TJA between 2019–2021 were analyzed at a single institution, and periprosthetic joint infection (PJI), wound drainage, 30 and 90-day emergency room (ER) readmission due to wound complications, aseptic loosening, and revision surgery rate were compared between patients undergoing intraoperative CHG versus betadine lavage prior to closure. Baseline demographics were controlled, and multivariate logistic regression was performed to compare complication rates.

**Results:**

A total of 410 TJA, including 160 hip and 250 knee arthroplasties were included. Compared to the dilute betadine cohort, all TJA patients undergoing CHG lavage had a statistically significant lower 30 and 90-day emergency room readmission rate due to wound complications. Both hip and knee arthroplasty patients with CHG had a statistically significant lower rate of postoperative superficial drainage and dressing saturation at clinic follow-up, but only knee arthroplasty patients had significant decreased readmission rate for incisional wound vacuum placement and close inpatient monitoring of wound healing. Among all TJA, there was no significant association in the rate of PJI requiring return to the OR between groups.

**Conclusions:**

Although betadine is cost-effective and has been shown to reduce PJI rates, there remains concerns in the literature over soft tissue toxicity and wound healing. This study suggests CHG may be as efficacious as dilute betadine in preventing PJI while also decreasing the risk of superficial drainage and wound complications needing unplanned ER visits during the acute postoperative period.

## Background

Surgical site infections (SSIs) represent a major concern for both surgeons and patients, especially in the setting of invasive surgeries like total joint arthroplasty (TJA). Periprosthetic joint infections (PJIs) following total joint replacements are of particularly high concern for orthopaedic surgeons given the cost, threat to patient health and outcome, and overall healthcare burden they encompass. The costs of these infections and their sequelae have been reported to be anywhere from $68,053 to $107,264 per case in the United States, resulting in a healthcare burden of greater than $1 billion annually [17, 19, 20, 24]. While PJI remains one of the most morbid complications after primary TJA with estimated yearly costs exceeding $250 million a year, recent literature has also emphasized the economic burden and importance associated with unplanned Emergency Department and Urgent Care visits. With the rising costs of healthcare and the aging population of individuals requiring TJA, these economic consequences cannot be ignored.

The number of PJI has already more than doubled from 0.92% to 2.07%, and by 2025, the projected number of PJI in the United States is estimated to be 650,000 with a cost rising to almost 1.44 billion per year [2, 13, 16, 22]. Historically, multiple measures have been taken to minimize incidence of surgical site infections in TJA including prophylactic antibiotics, laminar flow operating rooms, screening for infectious agents, draping, and skin sterilization [9, 21]. Betadine contains povidone-iodine that releases free iodine, which is toxic to microorganisms and has been shown to have broad-spectrum bactericidal activity with relatively minimal side effects to the wound [10]. Although Povidone-Iodine has been the standard for skin sterilization in TJA for the last decade, there have been concerns with its use as there is a continued presence of postoperative infection. Over the last decade there have been multiple recalls of Povidone-Iodine antiseptics due to concern over lack of sterility and bacterial contaminants. Chlorhexidine Gluconate (CHG) has been shown to be an effective bactericidal method for skin sterilization by binding to anionic molecules in bacteria cell wall, further decreasing microbial load in vitro when compared to Povidone-Iodine [7]. However, the research comparing these two solutions is largely pre-clinical and little work has been done to compare the effects post operatively [8, 9].

This study aims to compare the rate of one-year PJI, wound complications, and inpatient hospital readmissions in our total joint arthroplasty patients using either Chlorhexidine Gluconate or Povidone-Iodine as intraoperative lavages prior to skin closure. Due to prior studies suggesting the inactivation of iodine with serum and possible toxicity of iodine to fibroblasts and synovium, this study seeks to compare the effect of lavage solutions and wound healing rates [26]. We predict that patients treated with CHG will have lower rates of superficial drainage and wound-related hospital readmissions compared to the betadine cohort.

## Methods

This was a retrospective study performed at a single academic orthopaedic hospital and included patients receiving primary total knee arthroplasty (TKA) and total hip arthroplasty (THA) between 2019 and 2021. During the collection period there were a total of 410 primary TJA’s performed at our institution including 160 THA and 250 TKA that were available for analysis.

All patients received standardized preoperative optimization including weight control, glucose control, and medical co-management when indicated. On the date of surgery all patients were prepped and draped in a standardized fashion including preoperative shaving with electrical clippers as needed and scrub with CHG for skin antisepsis. Preoperative prophylaxis included weight based antibiotic dosing of Ancef, or Vancomycin and Gentamycin for those with penicillin allergies, or for those with a positive MRSA colonization. Post operatively, patients received 24 h of antibiotic prophylaxis with Ancef, or Vancomycin and Gentamycin. All patients received wound lavage prior to fascia skin closure; the use of betadine versus CHG was based upon surgeon preference. Author DS performed all TJA with Betadine lavage from 2019–2020 then switched to CHG from 2020–2021 once CHG became available at our institution. Authors SY and WM only used Betadine lavage from 2019–2021. Betadine lavage was prepared by mixing 17.5 mL of 10% povidone-iodine solution with 500 mL of sterile normal saline, while CHG was provided in the form of sterile packed 450 mL bottle Irrisept (Irrimax Corporation, Laerceville, GA). In both groups prior to fascia skin closure, the lavage was poured into the wound and let soak for 3 min prior to suctioning and irrigating the wound with 1L normal saline through a pulse lavage system. All patients received standard multilayered closure and standard dry dressings without negative pressure or drain placement.

These cases were retrospectively reviewed for rates of periprosthetic joint infection, wound drainage, 30 and 90-day emergency room readmission due to wound complications, aseptic loosening, and revision surgery. 30 and 90-day readmission rates for TJA were recorded based on need for inpatient monitoring of wound with incisional vacuum dressing placement from acute dressing saturation or wound dehiscence. Superficial drainage was recorded based on presence of Mepilex Ag dressing saturation at least 0.5cm^2^ for patients at postoperative clinic or emergency room visits not needing overnight readmission and readmitted patients who needed incisional vacuum placement. PJI was determined using the updated 2018 criteria for periprosthetic infections including presence of a sinus tract or two positive cultures with the same pathogen comprising the major criteria, and elevated CRP, D-dimer, ESR, synovial WBC, Leukocyte esterase, alpha-defensin, synovial PMN, synovial CRP comprising minor criteria [18]. Patients readmitted for revision surgery involving periprosthetic fractures, dislocation, loosening, and hardware failure not related to wound complications were recorded as revision surgery. All data were collected from the institution’s electronic medical record system.

### Statistical analysis

Our data were analyzed using Stata 17 (StataCorp LLC, College Station, TX). Continuous variables were described with mean ± standard deviation, and categorical variables were described as absolute and relative frequency. Descriptive statistics were utilized for patient characteristics. Continuous variables were compared using the Wilcoxon rank-sum test, and binary outcomes were compared using Chi-squared or Fisher exact test as appropriate. A multivariate bivariate logistic regression was performed to identify lavage solution as an independent risk factor for postoperative outcomes. The multivariate regression analysis was adjusted for performing surgeon, age, sex, smoking, BMI, ASA, and medical comorbidities to account for confounding variables. For all the tests, the significance level *P* < 0.05 was considered statistically significant.

## Results

A total of 410 patients with TJA were analyzed with 206 receiving Betadine irrigation and 204 receiving CHG. The mean age of the study population was 66.3 ± 11 and mean BMI of 30.3 ± 6.1. We observed a higher mean age with those receiving CHG 67.3 ± 9.8 compared to Betadine 65.1 ± 12.4, however this difference was not considered statistically significant (*p* = 0.05) (Table 1). There were no statistically significant differences with regard to performing surgeon, BMI, sex, type of arthroplasty, ASA classification, and smoking status between the two groups.

**Table 1:** Demographic data

Variable			Betadine	Irrisept	Total	*P*-value
Total		N	206	204	410	
Age		Mean (SD)	65.1 (12.4)	67.3 (9.8)	66.3 (11.1)	*p* = 0.050
BMI		Mean (SD)	30.0 (5.9)	30.6 (6.4)	30.3 (6.1)	*p* = 0.289
Sex	Male	N (%)	91 (49.7)	92 (50.3)	183	
	Female		115 (50.7)	112 (49.3)	227	*p* = 0.851
TJA	Hip	N (%)	86 (53.8)	74 (46.3)	160	
	Knee		120 (48.0)	130 (52.0)	250	*p* = 0.256
ASA	1	N (%)	3 (75.0)	1 (25.0)	4	
	2		96 (53.0)	85 (47.0)	181	
	3		102 (46.8)	116 (53.2)	218	
	4		5 (71.4)	2 (28.6)	7	*p* = 0.279
Smoking	No	N (%)	202 (50.8)	196 (49.2)	398	
	Yes		4 (33.3)	8 (66.7)	12	*p* = 0.234
Disposition	Home	N (%)	175 (49.0)	182 (51.0)	357	
	Other		31 (58.5)	22 (41.5)	53	*p* = 0.213

Periprosthetic joint infection was diagnosed within 90 days of surgery in five of those receiving betadine, and three of those receiving CHG (1.2% vs 0.7%, *p* = 0.39). At one year follow up, there were no further cases of PJI observed amongst all patients. Readmission for inpatient wound monitoring with incisional wound vac placement within one month of surgery was greater in the betadine cohort with 15 readmissions, compared to the CHG cohort with two readmission (3.7% vs 0.5%, *p* = 0.001) (Table 2). This trend continued with any wound-related readmission within three months (19 (4.6%) vs 6 (1.5%), *p* = 0.002). There were more cases of superficial drainage in the betadine cohort (30) compared to the CHG cohort (6) (7.3% vs 1.5% *P* < 0.001). Those found to have superficial drainage were treated with local wound care and incisional vacuum placement with some cases receiving antibiotics based on the treating physician’s discretion. All patients with superficial drainage were not deemed to have evidence of PJI via joint aspiration, and laboratory evaluation based on the updated 2018 PJI criteria [18].

**Table 2:** Complications for betadine versus CHG lavage: all total joint arthroplasty

Complication			Betadine	Irrisept	Total	*P*-value
Readmission within 30 days	No	N (%)	191 (46.6)	202 (49.3)	393 (95.9)	
	Yes		15 (3.7)	2 (0.5)	17 (4.1)	*p* = 0.001
Readmission within 90 days	No	N (%)	187 (45.6)	198 (48.3)	385 (93.9)	
	Yes		19 (4.6)	6 (1.5)	25 (6.1)	*p* = 0.002
Periprosthetic joint infection 90 days	No	N (%)	201 (49.0)	201 (49.0)	402 (98.0)	
	Yes		5 (1.2)	3 (0.7)	8 (2.0)	*p* = 0.391
Periprosthetic joint infection 1 year	No	N (%)	201 (49.0)	201 (49.0)	402 (98.0)	
	Yes		5 (1.2)	3 (0.7)	8 (2.0)	*p* = 0.391
Aseptic loosening	No	N (%)	205 (50.0)	204 (49.0)	409 (99.8)	
	Yes		1 (0.2)	0	1 (0.2)	
Superficial Drainage	No	N (%)	176 (42.9)	198 (48.3)	374 (91.2)	
	Yes		30 (7.3)	6 (1.5)	36 (8.8)	*p* < 0.001
Revision surgery	No	N (%)	196 (47.8)	200 (48.8)	396 (96.6)	
	Yes		10 (2.4)	4 (1.0)	14 (3.4)	*p* = 0.079

When comparing THA alone, the prevalence of superficial drainage was 11 in the betadine cohort, and one in the CHG cohort (6.9% vs 0.6%, *p* = 0.006) (Table 3). There were no observed differences found between readmission rates, or PJI. When comparing TKA alone, the prevalence of readmission within 1 month was statistically greater in the betadine cohort versus the CHG cohort (10 (4%) vs 1 (0.4%), *p* = 0.005) (Table 4). This trend continued for any readmission within three months, with 14 readmissions in the betadine cohort versus four readmissions in the CHG cohort (5.6% vs 1.6%, *p* = 0.004). Similar to the THA cohort, the TKA cohort had a statistically greater rate of superficial drainage in the betadine cohort compared to the CHG cohort (19 (7.6%) vs 5 (2%), *P* < 0.001). There were no observed differences in rates of PJI at three months and one year post operatively between the cohorts in those receiving TKA. Between the betadine and CHG cohorts, there were no differences observed amongst the 14 revision surgeries performed not related to infection nor the prevalence of aseptic loosening.

**Table 3:** Complication rates for betadine versus CHG lavage: total hip arthroplasty

Complication			Betadine	Irrisept	Total	*P*-value
Readmission within 30 days	No	N (%)	81 (50.6)	73 (45.6)	154 (96.3)	
	Yes		5 (3.1)	1 (0.6)	6 (3.8)	*p* = 0.079
Readmission within 90 days	No	N (%)	81 (50.6)	72 (45.0)	153 (95.6)	
	Yes		5 (3.1)	2 (1.3)	7 (4.4)	*p* = 0.611
Periprosthetic Joint Infection 90 days	No	N (%)	84 (52.5)	73 (44.6)	157 (98.1)	
	Yes		2 (1.3)	1 (0.6)	3 (1.9)	*p* = 0.416
Periprosthetic Joint Infection 1 year	No	N (%)	84 (52.5)	73 (45.6)	157 (98.1)	
	Yes		2 (1.3)	1 (0.6)	3 (1.9)	*p* = 0.483
Aseptic Loosening	No	N (%)	85 (53.1)	74 (46.3)	159 (99.4)	
	Yes		1 (0.6)	0	1 (0.6)	
Superficial Drainage	No	N (%)	75 (46.)	73 (45.6)	148 (92.5)	
	Yes		11 (6.9)	1 (0.6)	12 (7.5)	*p* = 0.006
Revision Surgery	No	N (%)	80 (50.0)	72 (45.0)	152 (95.0)	
	Yes		6 (3.8)	2 (1.3)	8 (5.0)	*p* = 0.191

**Table 4:** Complication rates for betadine versus CHG lavage: total knee arthroplasty

Complication			Betadine	Irrisept	Total	*P*-value
Readmission within 30 days	No	N (%)	110 (44.0)	129 (51.6)	239 (95.6)	
	Yes		10 (4.0)	1 (0.4)	11 (4.4)	*p* = 0.005
Readmission within 90 days	No	N (%)	106 (42.4)	126 (50.4)	232 (92.8)	
	Yes		14 (5.6)	4 (1.6)	18 (7.2)	*p* = 0.004
Periprosthetic Joint Infection 90 days	No	N (%)	117 (46.8)	128 (51.2)	245 (98.0)	
	Yes		3 (1.2)	2 (0.8)	5 (2.0)	*p* = 0.442
Periprosthetic Joint Infection 1 year	No	N (%)	117 (46.8)	128 (51.2)	245 (98.0)	
	Yes		3 (1.2)	2 (0.8)	5 (2.0)	*p* = 0.442
Aseptic Loosening	No	N (%)	120 (48.0)	130 (52.0)	250 (100.0)	
	Yes		0 (0)	0 (0)	0 (0)	
Superficial Drainage	No	N (%)	101 (40.4)	125 (50.0)	226 (90.4)	
	Yes		19 (7.6)	5 (2.0)	24 (9.6)	*p* < 0.001
Revision Surgery	No	N (%)	116 (46.4)	128 (51.2)	244 (97.6)	
	Yes		4 (1.6)	2 (0.8)	6 (2.4)	*p* = 0.265

## Discussion

With the new emphasis on value-based healthcare bundled payments, it is important for arthroplasty surgeons to optimize existing techniques used in the prevention of periprosthetic joint infections and wound complications to reduce hospital costs and improve patient satisfaction. In this study, the rate of PJI in the betadine and CHG group was less than 1.3%, which is consistent with the current literature and reflects the generalizability of our results [19]. While dilute betadine lavage has been previously shown to be safe, effective in reducing PJI compared to normal saline, there are limited studies examining the efficacy of CHG versus dilute betadine [2]. Dressing saturation, wound drainage, and need for urgent evaluation in the emergency room may increase patient dissatisfaction and are costly to the healthcare system [7, 20]. In this study, we found that CHG lavage may help decrease TKA 30 and 90-day hospital inpatient wound related readmissions and all TJA wound drainage complications compared to dilute betadine, but there was no significant difference in wound related readmissions for THA.

Due to betadine being safe, inexpensive, bactericidal against methicillin resistant S. Aureus, and readily available within most operating rooms, there are prior orthopaedic studies demonstrating betadine lavage efficacy in preventing infections compared to normal saline without adverse effects on wound healing, bone union, and clinical outcome [2–4]. However, there are existing concerns regarding not only the cytotoxic effects of iodine on renal and thyroid disease patients, but also local host soft tissue toxicity resulting in a possible increased rate of wound healing complications, including dehiscence and drainage [7, 11, 25]. Prior lab studies have suggested the possible toxicity of povidone on host fibroblasts and keratinocytes, and these findings warrant further investigation into the toxic effects on the joint capsule, synovium, and the physiologic environment needed for fascia healing, incisional subdermal healing, and prevention of infection [1, 5, 6, 12, 26].

The potential effects of povidone-iodine on fibroblasts and keratinocytes may explain the increased rate of superficial drainage and wound complications seen in our TKA and THA patients. Prior reports of betadine causing local allergic reactions and irritative contact dermatitis may also contribute to the increased superficial drainage and concern among our TJA postoperative patients requiring further unplanned emergency department (ED) evaluation [14]. Although none of our patients experienced serum iodine sensitivity causing metabolic acidosis, it is possible the local irritative contact dermatitis and inhibition of fibroblast cell growth lines may have interfered with wound healing speed and tissue regeneration leading to persistent drainage [1, 5, 6]. In our study, betadine lavage increased the risk of drainage requiring incisional wound vac placement to further enforce skin edge approximation, reduce edema, and stimulate perfusion in areas that may be adversely reactive to iodine.

CHG has been shown to have a strong affinity for binding to skin and mucous membranes, which is ideal for capsule and skin closures [15]. Skin preparations using povidone-iodine frequently emphasize the need for the solution to dry in order to reach its full anti-microbial potential, and molecular studies have shown that the activity of iodophors decline drastically after thorough rinsing with water [8, 14]. However, intraoperative dilute betadine lavage does not allow for a dry iodine solution, and a study has shown iodine may be inactivated by blood and serum, which makes the solution problematic for surgical wound lavage [14]. CHG does not have the same properties of being inactivated by blood, and has been shown to possibly stay longer and active in host soft tissue [8]. Especially in our TJA patients who have increased superficial drainage postoperatively, it is important during the capsule and retinacular closure that the wound healing environment is optimal after surgical trauma created during surgical releases and ligamentous balancing. Sobel et al. found that human patellar allografts soaked in CHG had no significant changes in tensile load or stiffness when compared to saline, which is important for arthroplasty surgeons to consider during closure of a TKA [23].

Our findings of increased superficial drainage for all TJA patients and inpatient readmissions in TKA patients receiving intraoperative dilute betadine lavage compared to CHG may have important financial implications. Although prior studies have examined the cost benefit relationship of a unit of betadine solution costing around $2.04 compared to a unit of CHG Irrisept solution at $60.00, it is important to consider the financial implications from our patients returning to the ED for unplanned wound complications. Sibia et al. reported that unplanned ED visits costed the hospital on average $429.00 per return visit and hospital costs per readmission were estimated to average around $6484.00 [20]. While the lavage solution had no difference in discharge destination or outcomes for THA, increased drainage seen in patients may lead to poor patient reported outcomes and increased risks of postoperative stiffness and arthrofibrosis. These postoperative TJA patients with wound complications are often advised to limit activity and knee flexion to allow for soft tissue rest and healing. Although there was no increased rate of revision surgery in our patients, our patients with superficial drainage were advised to temporarily stop physical therapy, decrease activity level, and limit knee flexion, which can lead to overall limited range of motion necessitating further manipulation under anesthesia, lysis of adhesions, and revision surgery. Although betadine lavage may cost less compared to Irrisept solution per unit, the overall decrease in rates of unplanned emergency room visits and incisional vacuum placement with CHG lavage should be considered when monitoring orthopaedic postoperative wounds containing metal implants.

However, there are limitations to consider in this study, including its retrospective design, nonrandomized nature, and lack of observer blinding of intraoperative lavage type which may limit the generalizability of our results. Due to the efficacy of betadine from prior reports, our institution over the past 4 years has used dilute betadine lavage instead of normal saline for primary TJA so we were not able to compare CHG and dilute betadine outcomes to a control group consisting of normal saline lavage. Throughout the years, there are inevitable migration of surgical practices, including surgical nuances such as implant vendor changes, suturing technique and material, soft tissue retractors, and postoperative dressing variability that have profound effects on infection potential that limit anything but randomized prospective studies [8]. Due to the low rates of PJI and complications seen in our study, it is possible that many outcomes deemed not statistically significant may not have been powered to elucidate associations due to the sample size.

## Conclusion

Although betadine is cost-effective and has been shown to reduce PJI rates, there remains concerns in the literature over soft tissue toxicity and wound healing. Unplanned ED visits for wound healing evaluation are not only costly but may affect patient dissatisfaction. This study suggests CHG may be as efficacious as dilute betadine in preventing PJI while also decreasing the risk of superficial drainage and wound complications needing unplanned ED visits during the acute postoperative period.

## Data Availability

The data and materials for this study are not currently available for public use, and are not associated with a data repository. The data for the study are available by the corresponding author upon request.

## Abbreviations

SSI: Surgical Site Infection
TJA: Total Joint Arthroplasty
PJI: Periprosthetic Joint Infections
CHG: Chlorhexidine Gluconate
TKA: Total Knee Arthroplasty
THA: Total Hip Arthroplasty
ED: Emergency Department
